# Undersaline ESD for Large Colorectal Lesions: A Narrative Review

**DOI:** 10.3390/medicina62101912

**Published:** 2026-10-03

**Authors:** Rodolfo Augusto Assis Rezende, Roberto de Sire, Antonio Di Martino, Davide Massimi, Ludovico Alfarone, Antonio Capogreco, Cesare Hassan, Alessandro Repici, Roberta Maselli

**Affiliations:** 1 Endoscopy Unit, Gastroenterology Department, IRCCS Humanitas Research Hospital, Via Alessandro Manzoni 56, 20089 Rozzano, Italy; rodolfo.assisrezende@humanitas.it (R.A.A.R.); roberto.desire@humanitas.it (R.d.S.); antonio.dimartino@humanitas.it (A.D.M.); davide.massimi@humanitas.it (D.M.); ludovico.alfarone@humanitas.it (L.A.); antonio.capogreco@humanitas.it (A.C.); cesare.hassan@hunimed.eu (C.H.); alessandro.repici@hunimed.eu (A.R.); 2 Department of Biomedical Sciences, Humanitas University, Via Rita Levi Montalcini 4, Pieve Emanuele, 20072 Milan, Italy; 3 Gastroenterology and Digestive Endoscopy Unit, Humanitas San Pio X, Via Francesco Nava 31, 20159 Milan, Italy

**Keywords:** colorectal neoplasms, endoscopic resection, undersaline submucosal dissection, underwater ESD

## Abstract

Endoscopic submucosal dissection (ESD) is the standard of care for the en bloc resection of large colorectal lesions but remains technically demanding, with long procedure times, a steep learning curve, and complication rates that have constrained adoption outside expert Eastern centres. Undersaline ESD (UESD) has emerged as a modification in which gas insufflation is replaced by immersion in isotonic saline, exploiting the distinct physical properties of a liquid-filled lumen to address several limitations of the conventional technique. Although frequently described as “underwater” ESD, the term “undersaline ESD” is more precise, since its advantages derive specifically from the ionic content of saline rather than from submersion alone. This narrative review synthesises the mechanistic rationale and the accumulating clinical evidence on UESD for large colorectal lesions. Saline immersion alters monopolar electrosurgical behaviour towards a coagulation-dominant profile, generates intrinsic traction on the mucosal flap through differential buoyancy, magnifies the dissection plane, and dissipates thermal energy. Across observational series and two recent meta-analyses, UESD is associated with faster submucosal dissection without compromising en bloc resection, R0 resection or short-term safety. The randomised evidence is not uniform: two of three trials reported faster dissection, while the largest found no difference in its primary endpoint, with lesion position determining the relative advantage of each technique. The benefits appear concentrated in specific settings such as submucosal fibrosis, gravity-side lesions, and, on preliminary data, non-expert operators, rather than distributed across all procedures. Key evidence gaps include the East Asian concentration of randomised data, the heterogeneity of technical implementation, and an absence of long-term oncological outcomes.

## 1. Introduction

Endoscopic submucosal dissection (ESD) was first developed in Japan in the late 1990s and early 2000s for the en bloc removal of early gastric cancer, where retrieval of the lesion in a single specimen permits accurate histopathological staging and reliable assessment of resection margins [1,2]. By overcoming the size constraints of conventional endoscopic mucosal resection (EMR), ESD achieves higher rates of en bloc and R0 resection and, in turn, lowers the likelihood of local recurrence [3]. Extension of the technique to the colorectum proved more challenging, owing to the thin, folded colonic wall, difficult access to the submucosal plane, and a heightened risk of perforation [1,4,5]. However, from the late 2000s onwards it was progressively adopted for large colorectal lesions, typically those ≥20 mm, for which piecemeal EMR carries a high risk of incomplete resection and recurrence. Adoption in Western countries lagged further, constrained by long procedure times, a steep learning curve, and higher adverse-event rates outside high-volume expert centres [6]. These limitations, which persist even as new knives and electrosurgical platforms have matured, have motivated a range of modified-ESD strategies such as the pocket-creation method, tunnelling, hybrid ESD, and traction-assisted approaches, each aiming to improve exposure of the dissection plane, accelerate the procedure, or reduce complications [4].

In parallel, underwater endoscopy emerged from a distinct line of development. Underwater colonoscope intubation gained favour for its comfort and improved adenoma detection rate [7], and the principle of operating within a saline-filled lumen was subsequently translated to therapeutic endoscopy in the form of underwater EMR [8,9]. Building on this foundation, ESD performed in a liquid-filled lumen was first described as underwater ESD, and was proposed to harness the optical and mechanical properties of this environment to address the specific shortcomings of conventional ESD (CESD). This remains the designation adopted by the ESGE, which recommends saline in preference to water as the immersion medium [10]. The recommendation is itself an acknowledgement that the medium is not incidental, as the effects of a liquid-filled lumen fall into two classes. The buoyancy of the mucosal flap, optical magnification, and heat dissipation follow from the bulk physical properties of any liquid, whereas the altered behaviour of monopolar electrosurgical currents depends on the dissolved Na^+^ and Cl^−^, which raise conductivity and change diathermic energy delivery in ways that may enhance haemostasis [11,12]. Because the latter underlies several of the distinctive advantages discussed below, the terminology used here specifies the medium, in line with designations such as the saline-immersion/irrigation technique [13,14,15]. The acronym UESD is therefore retained unchanged and denotes undersaline ESD, which is the same procedure designated in the primary literature and guidelines as underwater ESD; the two classes of effect are distinguished throughout Section 3. This review examines the mechanistic rationale, the accumulating clinical evidence and the current limitations of UESD for large colorectal lesions, aiming to identify where the evidence is robust, where it remains uncertain, and where future research is most needed.

## 2. Methods

References were identified primarily by backward citation chasing, with reference lists of key studies, guidelines and reviews on immersion techniques in ESD screened iteratively until no further eligible reports emerged. This was supplemented by targeted PubMed searches using specific terms as questions arose during drafting (“endoscopic submucosal dissection”, “underwater endoscopy”, “underwater ESD”, “saline-immersion technique”, and “colorectal ESD”) and by studies known to the authors from practice in the field. Searching was conducted up to 8 June 2026 and restricted to full-text articles published in English. No protocol-driven database search was performed, and complete retrieval of the literature is not claimed.

Reports were considered eligible if they addressed ESD performed with the lumen filled with saline or, in earlier reports, water, whether as the index technique or as a comparator, and reported procedural or clinical outcomes; or if they provided ex vivo, animal or bench data bearing on the electrosurgical, thermal or optical behaviour of the immersed field; or if they were guidelines or consensus documents addressing immersion techniques. Studies of non-colorectal ESD were considered only where they inform the mechanistic argument developed in Section 3, and are identified as such throughout. Single case and video reports were not used as sources of comparative outcome data, but were retained where they document a technical development or device relevant to the conduct of the technique. Conference abstracts without a corresponding full-text publication were not included. Where several reports addressed the same question, precedence was given to the most recent and methodologically robust; the final reference list was agreed by all authors.

No formal risk-of-bias instrument was applied, as this would imply a systematic methodology that was not undertaken. Primary evidence is weighted throughout by study design, with randomised trials and propensity-matched cohorts given precedence over unmatched and single-arm series. The pooled analyses cited are considered alongside rather than above their constituent studies, since these are also discussed individually, and are drawn on for summary estimates and heterogeneity assessments rather than as independent confirmation. Comparative series are discussed individually rather than aggregated, as some originate from the same centres and may therefore draw on overlapping patient cohorts. Reporting follows the SANRA framework for narrative reviews [16]; see Supplementary Table S1.

## 3. Physical Principles and Electrosurgery—Saline Immersion vs. CO_2_

### 3.1. Voltage, Power and Impedance

The behaviour of monopolar electrosurgical currents is governed by the relationship between impedance, voltage, and power. In conventional gas-insufflated endoscopy, the electrode is surrounded by a non-conductive medium and current passes only through tissue, so the high impedance sustains the peak voltages that generate the sparks required for cutting [12]. When CO_2_ is replaced by 0.9% saline, the conductive liquid medium creates a parallel low-resistance pathway that reduces impedance by approximately 99% in experimental models, irrespective of the electrosurgical mode used [12]. This near-complete collapse in impedance produces a marked reduction in peak voltage, falling well below the threshold required for spark formation and markedly reducing the cutting effect [12]. Concurrently, the electrosurgical generator compensates for the voltage drop by substantially increasing power delivery, up to the safety limit programmed into each mode, converting the additional energy into heat and amplifying the coagulation effect [12]. The net result is a shift from a predominantly cutting to a predominantly coagulating current, functionally resembling a soft-coagulation mode, using the same knife and the same generator settings. Histologically, this produces a deeper and wider zone of coagulation around the point of application, consistent with full-thickness coagulation of the vessel wall rather than its transection, and attributable both to the fall in impedance and to the wider dispersion of electrical charge in the conductive medium [12]. This concerns the coagulation zone in the tissue contacted by the electrode, and is distinct from the conduction of heat beyond it into adjacent and deeper layers, considered in Section 3.4. In clinical practice, this principle has been applied as prophylactic saline-immersion vessel pre-sealing during third-space endoscopy: by briefly switching from CO_2_ to saline immersion before dissecting across a large vessel, the knife tip can coagulate without cutting, eliminating the need for device exchange to coagulation forceps and reducing intraprocedural bleeding requiring retreatment [11,17]. The same low-voltage current also enables safe retreatment of actively bleeding vessels, since superficial coagulation without cutting avoids coagulation-driven perforation [11].

### 3.2. Buoyancy Effect

When the colonic lumen is filled with saline, the mucosal and submucosal layers experience an upward buoyant force driven largely by the low density of submucosal adipose tissue, whereas the heavier muscularis propria remains deep. This differential flotation naturally separates the dissection plane, lifting the mucosal flap away from the muscle layer and providing intrinsic traction that counteracts the effect of gravity [14]. In practical terms, this means the flap opens spontaneously during dissection, improving exposure of the submucosal space without reliance on repeated submucosal injection or external traction devices [14]. The effect is most pronounced when the lesion is positioned on the gravity side, where buoyancy and gravity act in concert: saline pushes the flap upward while gravity pulls the scope and instrument downward, creating optimal counter-traction. Conversely, when the lesion sits on the non-gravity side, buoyancy can work against the operator by closing rather than opening the flap, and air bubbles may accumulate around the lesion, making it harder to maintain full submersion [14,18].

### 3.3. Improved Visualisation: Zoom Effect and Halation Reduction

The optical advantages of a liquid-filled lumen have been recognised since the first description of underwater EMR, where it was observed that water immersion produces a natural magnification of the mucosal architecture, particularly useful for interrogating lesion borders before and after resection [8]. The underlying mechanism is the higher refractive index of saline (approximately 1.33) compared with air (1.00), which generates optical magnification when the endoscope operates within a liquid-filled environment [18]. Applied to ESD, this magnified image enhances delineation of tissue layers, submucosal vessels, and the dissection plane, enabling more precise knife placement during submucosal dissection [14,19]. Halation is the bright glare produced by light reflecting off a gas-distended, wet mucosal surface, which can obscure fine detail during conventional CO_2_-insufflated ESD; saline immersion helps eliminate this artefact, thus improving submucosal visualisation [18,20]. One practical disadvantage is the formation of air bubbles generated by electrosurgical activation, which can temporarily obscure the visual field, particularly when they become trapped within the distal cap [14,20].

### 3.4. Heat-Sink and “Frozen Effect”

**Figure 1:**
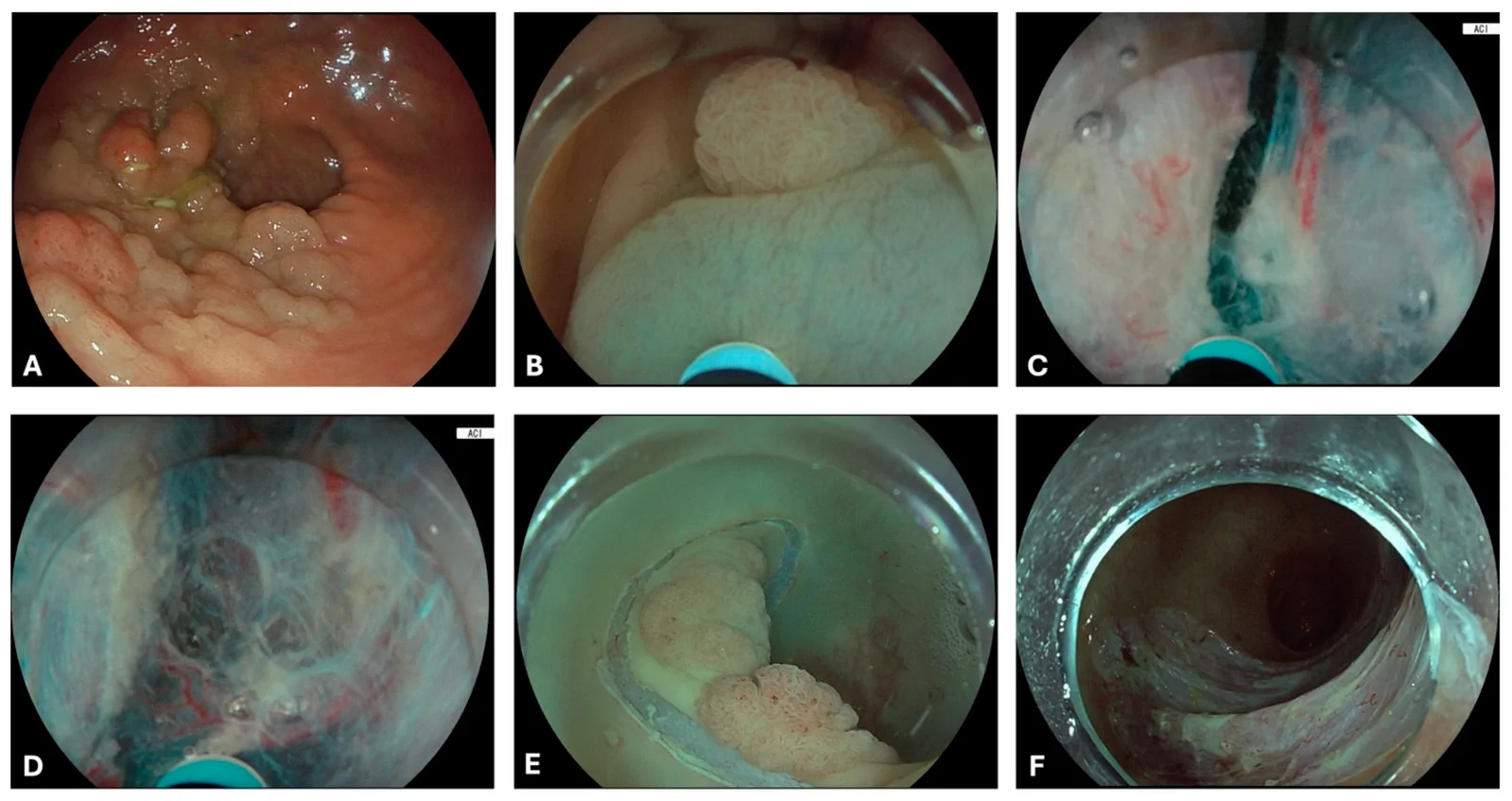
Procedural steps of UESD: endoscopic submucosal dissection of a large rectal laterally spreading tumour (LST). (**A**) An 80 mm rectal LST under white-light endoscopy with gas insufflation. (**B**) Mucosal incision with the lumen filled with isotonic saline; the lesion floats upward away from the muscularis propria. (**C**) Exposure of the submucosal plane during saline-immersed dissection, with the magnified, halation-free view characteristic of the technique. (**D**) Identification and pre-emptive coagulation of submucosal vessels under Amber-Red Colour Imaging (ACI). (**E**) Dissection at the midpoint, with the buoyant mucosal flap lifting to expose the submucosal layer. (**F**) The mucosal defect after complete en bloc resection.

Beyond its electrical properties, saline possesses high thermal conductivity and specific heat capacity, enabling it to absorb and dissipate heat generated by electrocautery [14]. This heat-sink effect limits the conduction of heat beyond the site of application, reducing collateral injury to the muscularis propria, which is the principal mechanism underlying post-ESD electrocoagulation syndrome (PECS) [18,21]. The coagulation dominance described in Section 3.1, combined with continuous saline irrigation during dissection, also produces a distinctive visual phenomenon during vessel pre-sealing: a frost-like whitening that spreads along the vessel wall and its branches, termed the “frozen effect”, which serves as real-time confirmation of successful coagulation before the vessel is transected. Together, these thermal properties contribute to a marked reduction in intraprocedural bleeding requiring haemostatic forceps reported in UESD series [13,17] (Figure 1).

## 4. Clinical Evidence

### 4.1. Technical Variants Under UESD

Studies described as UESD are not procedurally uniform. The most consequential variation concerns the co-application of the other modified-ESD strategies such as pocket creation, tunnelling and mechanical traction [10]. In saline-pocket ESD, pocket creation is intrinsic to the technique and applied to every case, so immersion and pocket creation are tested together [22]; the same holds for the single-arm cohort of Bosch and colleagues, in which the pocket-creation method was combined with immersion throughout [13]. Elsewhere, immersion is the constant and the remaining strategies are chosen case by case according to lesion morphology and access: in the cohort of de Sire and colleagues, circumferential incision, pocket creation, tunnelling and traction were each used in a minority of procedures and distributed comparably between the immersion and gas arms, leaving the medium as the only systematic difference [17]. Where a co-intervention is applied selectively and balanced across arms it is a source of variability rather than confounding; where it is applied universally to one arm alone, its effect cannot be separated from that of the medium.

UESD protocols differ in further aspects. Prophylactic saline-immersion vessel pre-sealing may be applied systematically, selectively, or not at all. Series labelled as the water-pressure method [23,24,25] emphasise waterjet pressure to open the submucosal plane, but dynamic adjustment of waterjet intensity for traction and visibility is described in series not using that label [17], so the designation could reflect nomenclature more than a distinct procedure.

### 4.2. Real-World Data

The first clinical experiences with UESD for colorectal lesions emerged from Japan in the late 2010s. Yoshii and colleagues reported a two-centre retrospective series of 40 colorectal neoplasms ≥20 mm or with submucosal fibrosis treated with a bipolar needle knife under saline immersion, achieving en bloc resection in all cases without perforation, delayed bleeding, or PECS [26]. The bipolar device was chosen to circumvent the dispersion of monopolar current in a conductive saline medium; this concern was subsequently addressed by Nagata in a single-operator series of 26 lesions, who showed that UESD could be performed safely and effectively with a standard monopolar knife, achieving 100% en bloc resection with no perforations and no electrolyte disturbances [27].

More recent Western data have refined the safety and efficacy profile of UESD, demonstrating outcomes that approach those of expert Eastern centres. In a propensity score-matched analysis of 82 non-pedunculated colorectal lesions (≥20 mm), de Sire and colleagues compared UESD, systematically combined with prophylactic saline-immersion vessel coagulation, against CESD. UESD was associated with a significantly higher R0 resection rate (97.6% vs. 82.9%; *p* = 0.02), faster dissection speed (31.8 vs. 22.9 mm^2^/min; *p* = 0.004), and an absence of intraprocedural bleeding requiring haemostatic forceps (0% vs. 14.6%; *p* = 0.002); the difference was most marked in non-expert operators, in whom R0 resection reached 100% compared with 80% with the conventional technique [17]. Comparable advantages emerged from a multicentre propensity score-matched analysis by Andrisani and colleagues comparing 100 Italian UESD cases with 400 CESD cases performed by expert Japanese endoscopists. UESD was associated with a higher en bloc resection rate (100% vs. 86.5%; *p* < 0.001) and a lower perforation rate (1% vs. 9.5%; *p* = 0.009), with dissection speed and R0 resection comparable to those reported in expert Eastern centres [19]. An important caveat is that the two arms were performed by different operators in different centres and countries and thus propensity matching cannot fully separate the contribution of the technique from that of operator and institutional factors. A further propensity-matched German cohort of 72 ESD procedures across the upper and lower gastrointestinal tract supported the safety and efficacy of UESD in an additional Western setting, with en bloc resection in 100% of cases in both groups and no perforations recorded in the UESD arm [28]. A single-centre Western experience by Bosch and colleagues reported outcomes of 181 large or complex colorectal lesions treated with saline-immersion ESD combined with the pocket-creation method, achieving en bloc resection in all cases and R0 and curative resection in 92.3% and 90.6%, respectively, with adverse events in only 4.4% [13]. Notably, over 93% of procedures were completed under conscious sedation, addressing one of the most cited practical barriers to broader Western adoption of colorectal ESD.

Complementary Eastern evidence comes from Kirita and colleagues, who extended these observations to a multi-operator real-world setting, comparing 92 matched pairs of UESD and CESD performed by seven endoscopists of varying experience. In this series the saline-immersion approach was implemented as the water-pressure method, a variant employing active waterjet pressure within the saline-filled lumen. Mean procedure time was significantly shorter with UESD (48.7 vs. 58.1 min; *p* = 0.032) without compromising en bloc or R0 resection rates or increasing perforation, and the benefit was preserved in both expert and non-expert subgroups, supporting the generalisability of the technique beyond highly selected single-operator series [23]. Two further Japanese propensity score-matched studies have highlighted additional clinical benefits aligned with the physical principles outlined above. Koyama and colleagues demonstrated that UESD was associated with a significantly lower incidence of PECS compared with CESD (0% vs. 11.1%; *p* = 0.027), findings consistent with the thermal-protective effect of saline immersion described in Section 3.4 [18]. Ozeki and colleagues addressed the specific challenge of submucosal fibrosis, comparing UESD with CESD in 133 fibrotic colorectal neoplasms, of which more than 70% exhibited severe fibrosis. The saline-immersion approach significantly shortened procedure time (median 43.5 vs. 72 min; *p* = 0.004) and was confirmed as an independent predictor of shorter dissection time on multivariate analysis, while en bloc resection and adverse-event rates remained comparable to the conventional technique [24].

### 4.3. Randomised Controlled Trials (RCTs)

Three single-centre randomised controlled trials have directly compared UESD and CESD for colorectal lesions, all confined to expert operators.

Harada and colleagues compared the combination of UESD with the pocket-creation technique against ESD with CO_2_ insufflation. The study enrolled 91 patients with laterally spreading tumours (LST) ≥20 mm. The combination of saline and pocket creation significantly accelerated submucosal dissection (median 20.1 vs. 16.3 mm^2^/min; *p* < 0.001) and shortened procedure time (29.5 vs. 41 min; *p* < 0.001), with en bloc and complete resection achieved in 100% of cases in both arms; the benefit was most pronounced for non-granular lesions and for lesions with fibrosis. Regarding safety outcomes, no perforations were recorded and overall adverse events were comparable between groups (8.9% vs. 8.7%) [22].

Nagata and colleagues [20] randomised 139 patients with colorectal lesions (median size 26 mm in the CESD arm and 25 mm in the UESD arm) to CESD or UESD performed by a single expert. The primary endpoint of dissection speed did not differ significantly between groups (median 19.9 vs. 17.4 mm^2^/min; *p* = 0.19), and en bloc resection was universal in both arms, without any perforations. On multivariate analysis, lesion positioning emerged as the only independent predictor of dissection speed (*p* < 0.001), with the non-gravity side favouring CESD and the gravity side favouring UESD, thus suggesting that the two techniques are complementary rather than competitive. The incidence of PECS did not differ significantly between techniques (UESD 2.9% vs. CESD 4.3%; *p* = 0.68), likely reflecting the trial’s limited power for this outcome.

A recent trial conducted by Oh and colleagues [29] randomised 56 patients with LSTs measuring 20–50 mm and showed a substantially shorter total procedure time (49.5 vs. 75.7 min; *p* = 0.001) and faster dissection speed (21.9 vs. 15.2 mm^2^/min; *p* = 0.001) with UESD, with universal en bloc and R0 resection and no perforations in either arm. The advantage of UESD was most evident in lesions ≥30 mm and in colonic (vs. rectal) locations. The benefits were most pronounced in lesions with submucosal fibrosis, in which procedure time was nearly halved (*p* < 0.001), consistent with Harada’s findings [22].

Across these three trials, en bloc resection was achieved in virtually all cases regardless of technique, and no perforations occurred in any arm, a consistent signal that UESD does not compromise oncological adequacy or short-term safety. Although intraprocedural bleeding was not a primary endpoint in any of these trials, the saline-immersion coagulation principle outlined in Section 3.1 has since been formally validated in a randomised controlled trial of prophylactic saline-immersion vessel pre-sealing during third-space endoscopy—both ESD and per-oral endoscopic myotomy (POEM)—which demonstrated a 57% per-patient and 79% per-vessel reduction in bleeding events requiring retreatment, along with a marked reduction in the need for instrument exchange to coagulation forceps [11]. These findings provide controlled-trial confirmation of the electrosurgical principles described above and support the rationale for saline-immersion pre-sealing for high-risk submucosal vessels during colorectal UESD. 

A distinction must be drawn between prophylactic vessel coagulation and the treatment of bleeding once it occurs. Under saline immersion, a few drops of blood are enough to cloud the pool and obscure the bleeding point, and the field can deteriorate faster than it would under gas [4,20]. Once bleeding has occurred, the view is better restored by exchanging than by removing fluid, through vigorous waterjet lavage with simultaneous aspiration or a dedicated perfusion system, with preliminary observations suggesting the latter remains effective during major bleeding [25]. Where the source can be identified, the coagulation-dominant low-voltage current allows it to be retreated with the knife itself, without exchange to haemostatic forceps [11,30]. Should the field remain obscured, aspirating the pool and reverting to CO_2_ insufflation permit conventional haemostasis before re-immersion [4,20,23].

### 4.4. Meta-Analyses

Two recent meta-analyses have synthesised the available comparative data on UESD for colorectal lesions.

Singh and colleagues [4] performed a pairwise meta-analysis in which seven studies (two randomised controlled trials [20,29] and five non-randomised studies), totalling 1401 patients (452 UESD, 949 CESD), were pooled. The authors concluded that UESD significantly shortens procedure time (SMD −1.33; 95% CI −2.34 to −0.32; *p* = 0.010) and accelerates dissection speed (SMD 1.01; 95% CI 0.35–1.68; *p* = 0.003) compared with CESD. En bloc resection (99% vs. 98%), R0 resection (98% vs. 93%), delayed bleeding, perforation, and PECS rates did not differ significantly between techniques. The PECS estimate trended in favour of UESD (4.4% vs. 10.4%; OR 0.38). Substantial statistical heterogeneity (I^2^ = 97% for procedure time, 87% for dissection speed) and the inclusion of largely observational data temper the strength of these conclusions [4].

The more recent network meta-analysis by de Sire and colleagues restricted inclusion to randomised controlled trials and provided the first formal indirect comparison between UESD and traction-assisted ESD (T-ESD), the principal alternative modified-ESD strategy. Four RCTs comprising 543 patients (98 UESD, 171 T-ESD, 274 CESD), all conducted in East Asia, were analysed. R0 resection rates were comparable across all three techniques (pooled estimates: UESD 99.1%, T-ESD 95.4%, CESD 95.5%), as were adverse-event rates. Dissection speed, however, was significantly higher with UESD than with both T-ESD (SMD 0.36; 95% CI 0.14–0.87) and CESD (SMD 0.54; 95% CI 0.15–0.92), whereas T-ESD did not differ significantly from CESD (SMD 0.18; 95% CI −0.15 to 0.50). SUCRA rankings placed UESD first for dissection speed (0.93), R0 resection (0.77), and safety (0.72). Among the modified-ESD techniques compared in this network, UESD was the only one with demonstrably superior procedural efficiency over CESD in randomised data [21]. Substantial statistical heterogeneity was found regarding the pooled outcomes for dissection speed (I^2^ = 87.9% for CESD, I^2^ = 36.9% for UESD and I^2^ = 96.2% for T-ESD), being lowest in the immersion arm, which suggests that much of the variability lies in how the comparator techniques were performed.

Substantial statistical heterogeneity attends the dissection-speed estimates in both syntheses. The constituent studies differ in lesion selection, particularly in the proportion with submucosal fibrosis, and in operator experience, ranging from single-expert trials to multi-operator series. Technical variants such as pocket creation or tunnelling, prophylactic vessel coagulation, and the use of different knives could also have influenced the outcome, but these are inconsistently reported in the primary studies and neither synthesis was able to explore them through subgroup analysis. The pooled estimates therefore describe immersion-based ESD as a family of related procedures performed across a wide range of case difficulties rather than a single technique with a stable effect size.

Taken together, these meta-analyses point in a consistent direction: UESD is associated with faster submucosal dissection without compromising oncological adequacy or short-term safety and delivers an efficiency advantage that traction-based modifications have not matched in randomised comparisons. That the direction persists across studies of mixed design and across an RCT-only network synthesis is reassuring, although the magnitude of the advantage remains poorly defined, and the geographic concentration of randomised evidence in East Asia and the limited number of head-to-head trials remain important gaps.

The comparative clinical evidence discussed throughout this section is summarised in Table 1.

## 5. Future Directions

Despite the accumulating evidence supporting UESD as a safe and effective modification of CESD, several knowledge gaps warrant prospective investigation.

First, the randomised evidence base remains geographically narrow, as all RCTs comparing UESD with CESD have been conducted at expert East Asian centres, and thus confirmatory trials in Western settings would enhance generalisability. Western propensity-matched data suggest that the benefits of UESD may be greatest in non-expert operators and in settings where CESD has historically lagged behind Eastern benchmarks [17], but this hypothesis requires prospective validation.

Second, future research should clarify how UESD interacts with other modified-ESD strategies, and in particular whether UESD and traction-assisted ESD are best regarded as competing alternatives or as complementary components of a unified approach. The case for complementarity is mechanistically grounded: subgroup analyses of the Nagata trial [20] showed that UESD outperformed CESD for gravity-side lesions, where buoyancy and gravity act synergistically to open the mucosal flap, but CESD retained an advantage on the non-gravity side, where fluid collection and air-bubble accumulation could undermine the saline-immersion approach. Mechanical traction, which operates independently of lesion orientation, could in principle restore the UESD advantage in unfavourable positions, and combining the two may yield additive procedural benefits, particularly for complex lesions or anatomically challenging locations [17]. Direct head-to-head comparisons between UESD and traction-assisted ESD, as well as factorial trials evaluating their combination, would clarify whether an integrated hybrid strategy outperforms either modified technique alone [31]. Beyond traction, technical refinements such as continuous low-pressure saline perfusion, especially when synchronised with the electrosurgical unit and combined with optimised high-frequency settings, could reduce bubble formation, thereby improving visualisation and accelerating dissection, although this requires validation in larger studies [32].

Third, the current UESD literature is undermined by considerable technical heterogeneity. Published series differ in the extent of saline immersion (continuous throughout the procedure versus restricted to the dissection phase versus saline-pocket variants), in the type of knife employed (monopolar versus bipolar), in electrosurgical settings, and in whether prophylactic saline-immersion vessel pre-sealing is performed systematically or selectively. Emerging device innovations may further refine the technique: a recent propensity-matched study of a thin-needle knife with integrated high-pressure waterjet showed significantly faster dissection speeds in UESD, with the benefit most pronounced in non-expert operators and difficult-to-treat lesions [30]. Standardisation of the technical components, as well as comparative studies of these variants, would help identify which elements drive the observed efficacy gains and would facilitate reproducibility across centres.

The volume of saline instilled, and how the intraluminal pool is regulated during dissection, remains unstandardised. Reported figures are heterogeneous, but much of this variation reflects differing measurement conventions rather than disagreement on technique. In the UESD with pocket-creation method trial by Harada and colleagues, the saline volume used was modest (median 200 mL, range 120–250 mL), higher than the 150 mL used in the standard-ESD comparator [22]. At the other extreme, Masunaga and colleagues recorded a median cumulative injection of 2312 g (range 1234–13,866 g) per procedure when a continuous liquid-suction catheter attachment was employed. The latter figure represents total throughput rather than a static pool, since a near-identical volume (median 2121 g) was simultaneously aspirated, leaving net distension low [25]. The operative principle is therefore one of dynamic balance rather than a fixed maximum: saline is continuously instilled and drained to preserve the visual field while preventing overdistension. Most commonly this balance is maintained through the scope’s own working channel, with intermittent suction during dissection. Dedicated drainage systems offer an alternative, particularly when continuous outflow is desired: the Asclepius tube targets a steady-state luminal volume of approximately 500 mL through continuous low-flow suction [13] while simpler trans-anal or Foley-catheter arrangements have been used for distal lesions [33]. No prospective work has yet defined an optimal target volume or instillation rate, and the standardisation of these parameters is a reasonable priority for future technical study.

Saline immersion carries a plausible risk of clinically significant fluid absorption in patients with renal or cardiac comorbidity, in whom excretion of the absorbed sodium and water load is limited or tolerance of volume expansion reduced. During prolonged UESD, absorption is mechanistically plausible across the exposed submucosa of a large defect and, where a deep mural breach exists, by extravasation. Direct data are lacking, but the concern is established in hysteroscopic surgery, where a closed, richly vascularised cavity is distended under pressure and absorbed isotonic saline may precipitate hypervolaemia, pulmonary oedema and cardiac decompensation. The joint British and European Society for Gynaecological Endoscopy sets an upper fluid-deficit limit of 2500 mL for isotonic solutions, with lower thresholds in elderly patients and those with cardiovascular or renal comorbidity [34]. Comparable precautions are reasonable pending endoscopic data: baseline assessment of renal function and cardiac status, with deferral and cardiology input in decompensated heart failure; the lowest effective immersion volume, instilled at low pressure with continuous drainage; explicit recording of instilled and recovered volumes; and a lower threshold for conversion to gas insufflation as the procedure lengthens. Whether absorption is clinically relevant during UESD remains unknown, and fluid deficit would be a useful parameter to record in future series.

Thermoregulation during prolonged immersion is a related and almost entirely unexamined consideration. Procedures using large saline volumes over the one-to-two-hour timescale typical of colorectal UESD carry a theoretical risk of heat loss, and the wider endoscopic-surgery literature links room-temperature irrigation to measurable falls in core temperature [35]. Masunaga and colleagues used warmed saline as routine [25], yet no study in the UESD setting has formally assessed patient temperature or the value of fluid warming. Whether warmed irrigation confers a clinically meaningful benefit during prolonged immersion procedures is an open question well suited to future investigation.

Finally, the available evidence has focused almost exclusively on intraprocedural metrics such as dissection speed, en bloc and R0 resection, and short-term adverse events, while longer-term outcomes remain largely unexplored. Data on local recurrence, surveillance findings after curative resection, and the rate of conversion to surgery for histologically non-curative cases are essentially absent from the UESD literature. Future trials should incorporate these endpoints to allow UESD to be positioned more precisely within the broader treatment algorithm for large non-pedunculated colorectal lesions, and to confirm whether its procedural advantages translate into durable oncological benefit.

## 6. Conclusions

UESD represents a mechanistically coherent evolution of conventional colorectal ESD, in which the physical properties of a saline-filled lumen, such as altered electrosurgical behaviour, differential buoyancy of the submucosal plane, optical magnification, and thermal dispersion, translate into reproducible procedural advantages. Across observational series, expert-centre randomised controlled trials, and two recent meta-analyses, the technique is associated with faster submucosal dissection without compromising en bloc resection, R0 resection, or short-term safety. However, the association is not uniform across the randomised evidence, since the trial by Nagata and colleagues found no difference in overall dissection speed and identified lesion position as the only independent predictor of that outcome, so that the two techniques are better regarded as complementary than competing. The advantage of immersion appears concentrated where conventional ESD is most demanding: submucosal fibrosis, gravity-side lesions, and, on preliminary Western data requiring prospective confirmation, with non-expert operators. Important uncertainties remain, particularly the East Asian concentration of randomised data, technical heterogeneity across centres, and the near-absence of long-term oncological outcomes. Nonetheless, the convergence of mechanistic rationale and consistent procedure advantages supports UESD as a useful addition rather than a replacement within the colorectal ESD armamentarium, according to lesion location and operator experience.

## Figures and Tables

**Table 1:** Summary of comparative clinical evidence for undersaline ESD in colorectal lesions. Outcomes are presented as UESD vs. comparator where applicable.

Study, Year and Design	Lesions (n) and Features	En Bloc/R0	Efficiency (Time; Speed)	Safety
Early feasibility (single-arm)				
Yoshii et al., 2018 [26]; two-centre retrospective	n = 40; ≥20 mm; bipolar needle knife	100%/— (curative 87.5%)	Time 72.5 min	No perforation, delayed bleeding, or PECS
Nagata et al., 2018 [27]; single-operator series	n = 26; colorectal; monopolar knife	100%/—	Speed 10.4 mm^2^/min	No perforation; no electrolyte disturbance
Real-world comparative studies				
de Sire et al., 2025 [17]; PSM; UESD vs. CESD	n = 82; non-pedunculated ≥ 20 mm	R0 97.6% vs. 82.9% (*p* = 0.02)	Speed 31.8 vs. 22.9 mm^2^/min (*p* = 0.004)	Bleeding requiring forceps 0% vs. 14.6% (*p* = 0.002)
Andrisani et al., 2025 [19]; multicentre PSM; Western UESD vs. expert Japanese CESD	n = 100 vs. 400; colorectal	En bloc 100% vs. 86.5% (*p* < 0.001)/R0 98% vs. 98.3%	Speed 35.3 vs. 36.9 mm^2^/min (*p* = 0.53)	Perforation 1% vs. 9.5% (*p* = 0.009)
Ayoub et al., 2025 [28]; PSM; upper + lower GI	n = 72; mixed GI	En bloc 100% vs. 100%/R0 94.4% vs. 91.7%	Time 77 vs. 75 min (*p* = 0.08)	No perforation in UESD arm
Bosch et al., 2026 [13]; single-centre, single-arm; UESD + pocket creation	n = 181; large/complex colorectal	En bloc 100%/R0 92.3%	Speed 9 mm^2^/min (IQR 4.9–13.6)	Overall AEs 4.4%
Kirita et al., 2025 [23]; PSM; UESD vs. CESD	n = 92 matched pairs; colorectal	En bloc 100% vs. 100%/R0 92% vs. 96% (*p* = 0.536)	Time 48.7 vs. 58.1 min (*p* = 0.032)	Perforation comparable
Koyama et al., 2023 [18]; PSM; UESD vs. CESD	n = 205; colorectal	En bloc 100% vs. 98.4%/R0 100% vs. 91.9% (*p* = 0.05)	Speed 10.9 vs. 6.9 mm^2^/min (*p* < 0.001)	PECS 0% vs. 11.1% (*p* = 0.027)
Ozeki et al., 2021 [24]; PSM; UESD vs. CESD	n = 133; fibrotic (>70% severe)	En bloc 100% vs. 98.7%/R0 94.4% vs. 92.4%	Time 43.5 vs. 72 min (*p* = 0.004); independent predictor on MV	Overall AEs comparable
Randomised controlled trials				
Harada et al., 2019 [22]; UESD + pocket creation vs. CESD (CO_2_)	n = 91; LST ≥ 20 mm	En bloc 100% vs. 100%	Time 29.5 vs. 41 min (*p* < 0.001); speed 20.1 vs. 16.3 mm^2^/min (*p* < 0.001)	No perforation; overall AEs comparable (8.9% vs. 8.7%)
Nagata et al., 2025 [20]; UESD vs. CESD (single expert operator)	n = 139; median 25–26 mm	En bloc 100% vs. 100%	Speed 19.9 vs. 17.4 mm^2^/min (NS; *p* = 0.19)	No perforation; post-ESD bleeding 0% vs. 2.9%; PECS 2.9% vs. 4.3% (*p* = 0.68)
Oh et al., 2024 [29]; UESD vs. CESD (single expert operator)	n = 56; LST 20–50 mm	En bloc/R0 100% vs. 100%	Time 49.5 vs. 75.7 min (*p* = 0.001); speed 21.9 vs. 15.2 mm^2^/min (*p* = 0.001)	No perforation; delayed bleeding comparable; PECS 3.6% vs. 14.3% (*p* = 0.152)
Meta-analyses				
Singh et al., 2025 [4]; pairwise MA (2 RCTs + 5 observational)	n = 1401 (452 UESD/949 CESD); colorectal	En bloc 99% vs. 98%; R0 98% vs. 93%	Time SMD −1.33 (−2.34 to −0.32; *p* = 0.010); speed SMD 1.01 (0.35–1.68; *p* = 0.003)	Bleeding/perforation/PECS comparable; PECS 4.4% vs. 10.4% (OR 0.38)
de Sire et al., 2026 [21]; network MA (4 RCTs)	n = 543 (98 UESD/171 T-ESD/274 CESD); colorectal, all East Asia	R0: UESD 99.1%/T-ESD 95.4%/CESD 95.5%	Speed: UESD > CESD SMD 0.54 (0.15–0.92); UESD > T-ESD SMD 0.36 (0.14–0.87); T-ESD vs. CESD NS	AEs comparable

AE, adverse event; UESD, undersaline ESD; CESD, conventional ESD; GI, gastrointestinal; IQR, interquartile range; LST, laterally spreading tumour; MA, meta-analysis; MV, multivariate analysis; NS, not significant; OR, odds ratio; PECS, post-ESD electrocoagulation syndrome; PSM, propensity score-matched; SMD, standardised mean difference; T-ESD, traction-assisted ESD. — denotes not applicable or not reported.

## Data Availability

No new data were created or analyzed in this study. Data sharing is not applicable to this article.

## Supplementary Materials

The following supporting information can be downloaded at https://www.mdpi.com/article/10.3390/medicina62101912/s1: Table S1: Assessment of this narrative review against the Scale for the Assessment of Narrative Review Articles (SANRA).

## Abbreviations

The following abbreviations are used in this manuscript:

ACI	Amber-Red Colour Imaging
CESD	conventional endoscopic submucosal dissection
T-ESD	traction-assisted endoscopic submucosal dissection
LD	linear dichroism
UESD	undersaline ESD
EMR	endoscopic mucosal resection
ESD	endoscopic submucosal dissection
LST	laterally spreading tumour
PECS	post-ESD electrocoagulation syndrome
POEM	per-oral endoscopic myotomy
RCT	randomised controlled trial
R0	microscopically margin-negative resection
SMD	standardised mean difference
SUCRA	surface under the cumulative ranking curve
