# Supplementary material for: Undersaline ESD for Large Colorectal Lesions: A Narrative Review
Source: Medicina (Kaunas). 2026 Oct 3;62(10):1912. doi: 10.3390/medicina62101912 (PMC13707086; doi:10.3390/medicina62101912)
Supplement: Supplementary file 1 [file medicina-62-01912-s001.zip › medicina-4480509-supplementary.pdf]

**Table S1.** Assessment of this narrative review against the Scale for the Assessment of Narrative Review Articles (SANRA).

| SANRA item | Rating | Location | Basis for the rating |
| --- | --- | --- | --- |
| 1. Justification of the article's importance for the readership | 2 | Introduction | The limitations of conventional colorectal ESD that motivate modified techniques are stated explicitly. |
| 2. Statement of concrete aims or formulation of questions | 2 | Introduction, final paragraph | Four concrete aims are stated, each mapped to a designated section. |
| 3. Description of the literature search | 2 | Section 2 | Identification method, search terms, cutoff date, language restriction and eligibility criteria are specified. No protocol-driven search was undertaken, and this is stated. |
| 4. Referencing | 2 | Throughout | Key statements are supported by primary references at the point of assertion. |
| 5. Scientific reasoning | 2 | Sections 3–4; Table 1 | Evidence is weighted by study design, with discordant findings and design-specific limitations reported. |
| 6. Appropriate presentation of data | 2 | Section 4; Table 1 | Absolute rates are given for both arms, and pooled estimates with 95% confidence intervals. |

Ratings were completed by the authors using the published SANRA instrument. Each item is rated 0–2 according to the anchors specified in the instrument. The table indicates where in the manuscript each item is addressed; the sum score is not reported, as the scale is intended to support appraisal by editors and reviewers rather than self-certification by authors.
